# A Flexible Hybrid Quantum-classical Training Framework of Organ-at-Risk and Tumor Segmentation Models for Radiation Therapy Planning

**DOI:** 10.1038/s41598-026-40127-z

**Published:** 2026-02-16

**Authors:** Qian Sun, Jiale Chen, Yuqing Fan, Xiaofei Kong, Hao Jiang, Lei Li, Mengqing Wang, Aili Xuan, Xiaoguang Yang

**Affiliations:** 1 Department of Radiology, The First Affiliated Hospital of Bengbu Medical University, Bengbu, 233004 China; 2 Origin Quantum Computing Technology (Hefei) Co., Ltd., Hefei, 230088 China; 3 Department of Pediatrics, The First Affiliated Hospital of Bengbu Medical University, Bengbu, 233004 China; 4 Joint Research Center for Regional Diseases of IHM, Bengbu Medical University, Bengbu, 233030 China; 5 Hefei Benyuan Quantum Computing and Data Medicine Institute, Hefei, 230088 China

**Keywords:** Medical image segmentation, Computed tomography, Artificial intelligence, Quantum computing, Cancer, Computational biology and bioinformatics, Engineering, Mathematics and computing

## Abstract

Deep learning-based Organ-at-Risk (OAR) and tumor segmentation is vital for radiation therapy planning but often suffers from over-parameterization, requiring large datasets to avoid overfitting, which is impractical in small-sample medical settings. Traditional trainable parameter reduction methods, relying on structural lightweighting or low-rank approximation, may artificially limit model expressiveness and hurt performance. We propose a Hybrid Quantum-Classical Training Framework (HQC-TF) based on the Quantum Parameter Generation (QPG) technique to reduce trainable parameters while preserving model structure and adaptively determining parameter matrices’ ranks during training. This retains representational flexibility with parameter efficiency. HQC-TF uses independent Variational Quantum Circuits (VQCs) per channel, preserving channel independence and applying flexibly to deep neural network training. Experiments showed it significantly improved segmentation with fewer parameters compared to the classical training framework: UNetPP gained 6.77% IoU and 3.09% DSC for kidney tumors. Notably, it operates only during training via shallow quantum circuits, making it a practical, scalable solution for near-term clinical use in radiation therapy.

## Introduction

Organ-at-Risk (OAR) and tumor segmentation are pivotal for radiation therapy planning, as accurate delineation directly guides dose delivery and minimizes healthy tissue damage^1^. Deep learning has demonstrated notable advantages over traditional methods in broader cancer diagnosis and treatment^2–4^, with its potential being particularly prominent in the core radiotherapy planning task of OAR/tumor segmentation^5,6^. Specifically, recent advances in deep learning-based OAR and tumor segmentation models hold substantial promise for developing automated, rapid, and high-accuracy radiation therapy systems^7,8^. Despite these advancements, state-of-the-art models such as TransUNet (41.4M parameters)^9^ and MedSAM (93.7M parameters)^10^ exhibit severe over-parameterization. This issue is further compounded by the mismatch between their large trainable parameter counts and the scarcity of high-quality labeled OAR/tumor imaging data, representing a critical bottleneck for clinical translation. Deep learning models rely on sufficient labeled samples to constrain parameter optimization. When the number of trainable parameters far exceeds the information capacity of available data, the model cannot learn generalizable anatomical and pathological features of OARs and tumors. Instead, it tends to overfit noise, such as annotation inconsistencies and individual-specific trivialities in the limited training set, as the sparse data fail to provide adequate constraints for tuning millions of parameters. This creates a critical clinical bottleneck. Fine-tuning these over-parameterized models requires massive labeled datasets to avoid overfitting, yet OAR/tumor annotation remains extremely arduous. Key challenges include ambiguous organ boundaries, irregular tumor morphologies, and subtle pathological-healthy tissue distinctions. The inherent tension between high model complexity (large parameter counts) and low data availability thus directly amplifies over-parameterization, making it a core barrier to clinical translation. Reducing trainable parameters without sacrificing segmentation performance has thus become an urgent unmet need.

Existing solutions parameter reduction fall into two categories, both with inherent limitations. Structural lightweighting includes pruning^11^, knowledge distillation^12^, and quantization^13^. These techniques compress pre-trained models but inevitably degrade expressive power, hindering performance on complex medical tasks. Parameter Efficient Fine-Tuning (PEFT) originated in the field of natural language processing^14^ and avoids structural changes by only training a subset of parameters. According to the classification proposed by Xin et al.^15^, PEFT encompasses Addition-based Tuning and Partial-based Tuning. Addition-based Tuning includes representative methods such as Adapter Tuning^14^, Prompt Tuning^16^, Prefix Tuning^17^ and Side Tuning^18^. Partial-based Tuning covers Specification Tuning and Re-parameter Tuning^19^. Among these approaches, LoRA^19^ is a typical Re-parameter Tuning method that has proven effective in skin lesion, polyp, and brain tissue segmentation^20,21^. However, it requires explicit pre-definition of parameter matrix ranks, trading flexibility for efficiency, as higher ranks improve performance but negate the benefit of parameter savings.

Quantum Parameter Generation (QPG)-based Quantum-Train^22,23^ has emerged as a compelling alternative, leveraging Variational Quantum Circuits (VQCs) to generate neural network parameters without the need for pre-specified ranks. However, this approach suffers from two critical drawbacks: (1) it adheres to a purely quantum training paradigm, precluding flexible integration with classical training frameworks and thus failing to capitalize on their complementary advantages; (2) it deploys a single VQC for all parameter generation, breaching the principle of channel independence—a foundational requirement for feature extraction, as exemplified by the independence of heads in multi-head attention^24^ and convolution kernels^25^. The Born Rule^26^ enforces amplitude normalization, which induces cross-channel parameter correlation and thereby degrades training performance.

To address these gaps, we propose the Hybrid Quantum-Classical Training Framework (HQC-TF), a flexible paradigm tailored for OAR and tumor segmentation. Key design choices directly target existing limitations: (1) hybrid training allows arbitrary modules to be trained via classical or quantum methods, combining their respective advantages; (2) independent VQCs generate parameters for each channel, preserving kernel independence while inherently achieving weight normalization-like effects via the Born Rule^26^ and eliminating explicit normalization overhead; (3) adaptive parameter matrix rank determination avoids LoRA’s pre-definition constraint. Given the immaturity of real quantum hardware^27^, we use quantum simulation—an accessible and practical choice for Noisy Intermediate-Scale Quantum (NISQ) era research^28,29^.

Our key contributions are threefold:

1. We propose a flexible training framework tailored for OAR and tumor segmentation models, which adopts a hybrid quantum-classical training paradigm. Extensive experiments validate its effectiveness across five mainstream OAR and tumor segmentation architectures.
2. We develop two novel convolutional layers trained via QPG, namely Quantum Parameter Generation trained Convolution (QPGConv) and Quantum Parameter Generation Adaptation trained Convolution (QPGAConv). These layers not only minimize the required number of qubits while retaining channel independence but also inherently exhibit weight normalization-like properties, eliminating the need for explicit computation by leveraging the Born Rule^26^.
3. Our experiments confirm that the hybrid quantum-classical training paradigm exhibits outstanding versatility, scalability, and superior performance. Notably, as it only requires deployment during the training phase and relies on shallow quantum circuits, it demonstrates substantial potential for practical implementation in the NISQ era.

## Methodology

### Variational quantum circuit (VQC)

VQC can be regarded as an analog of the neural network in the field of quantum computing, and it is essentially a quantum circuit with trainable parameters. Whether the VQC performs computations on a quantum computer or a quantum simulator, its parameter optimization still relies on classical algorithms running on classical computers, such as the gradient descent algorithm.Quantum computing is fundamentally based on unitary operations, and the arbitrary implementation of these operations on quantum hardware poses certain complexities. However, any unitary operation can be decomposed into a series of 1-qubit rotation gates and 2-qubit entanglement gates. If a set of basic gates that can span the entire Hilbert space is selected, universal quantum computing can be achieved by implementing this set of basic gates on quantum computers.

**Fig. 1:**
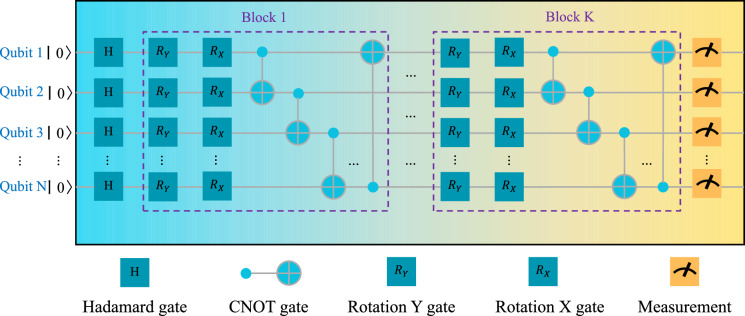
The Illustration of the Quantum Circuit We Designed. Like neural networks, the variational part of the quantum circuit consists of repeated blocks; as the number of blocks increases, both the representational capacity and the parameter count of the circuit increase accordingly. Longitudinally, the circuit is composed of N qubits, while transversely, the variational structure consists of K repeated strongly-entangled blocks. Qubits are all initialized to zeros. RX and RY all carry parameters, representing rotations around the X-axis and Y-axis respectively. The H gate is the Hadamard gate, which puts qubits into a superposition state. The CNOT gate is the controlled NOT gate, used for entangling qubits. Measurement operations are used to obtain the state of qubits.

Combined with qubits and measurement operations, quantum gates constitute a quantum circuit. Measurement is the process that induces wave function collapse, which transforms the superposition states of the qubits into definite classical bits. This is the only way to extract information from quantum states. Rotation gates are often used to embed classical data into quantum states. The structure of the quantum circuit we designed is illustrated in Fig. 1.

1 $$\begin{aligned} {|{\psi _{\text {final}}}\rangle } = \left( \prod _{k=1}^{K} \left[ \bigotimes _{j=1}^{N} \text {CNOT}_{j,j+1}^k \cdot \bigotimes _{i=1}^{N} R_X(\theta _X^{ki}) \cdot \bigotimes _{i=1}^{N} R_Y(\theta _Y^{ki}) \right] \right) \cdot \bigotimes _{i=1}^{N} H {|{0}\rangle }^{\otimes N} \end{aligned}$$

In our quantum circuit design, we employed the Hadamard (H) gate, rotation Y ($R_Y$) gate, rotation X ($R_X$) gate, and the Controlled Not (CNOT) gate, where $R_Y$ gate and $R_X$ gate are with parameters. The Hadamard gate is a single-qubit gate that transforms the state ${|{0}\rangle }$ into a uniform superposition state $\frac{{|{0}\rangle } + {|{1}\rangle }}{\sqrt{2}}$. The $R_Y$ and $R_X$ gates represent rotations around the Y-axis and X-axis of the Bloch sphere with angles as trainable parameters, respectively. The CNOT gate is used to create entanglement between two qubits, where the first qubit acts as the control qubit and the second qubit is the target qubit. Formally, the structure of our quantum circuit can be described by Eq. (1).

### Quantum parameter generation (QPG)

QPG uses VQCs that generate parameters for parts of a neural network during the hybrid quantum-classical training process. VQCs have their own parameters, which are essentially the angles of the quantum rotation gates. These parameters are trainable, whereas the parameters of the neural network are not directly trained; instead, they are obtained through QPG. This design reduces the total number of trainable parameters.

QPG is solely responsible for generating the weights of the neural network, and it receives information through backpropagation. This design eliminates the need for an explicit encoding operation within the VQCs, which is a significant advantage that we further discuss in the discussion section.

QPG updates its parameters by functioning as an additional branch in the neural network architecture. This is similar to side-tuning^18^, yet there are differences: QPG only exerts an effect on the parameter updates of the neural network. Formally, we denote the parameters generated by the QPG as $\boldsymbol{\theta }_{g}$, and the parameters of the QPG itself as $\boldsymbol{\theta }_{q}$. The QPG can be represented as a function $g$, while the neural network is represented as a function $f$. We further denote the input to the neural network as $\textbf{x}$, and the output of the neural network as $\textbf{y}$. The relationship between these components is described in Eq. (2).

2 $$\begin{aligned} \left\{ \begin{aligned} \textbf{y}&= f(\boldsymbol{\theta }_{g}, \textbf{x}) \\ \boldsymbol{\theta }_{g}&= g(\boldsymbol{\theta }_{q}) \end{aligned} \right. \end{aligned}$$

If the asymptotic complexity of $\boldsymbol{\theta }_{g}$ is $O(n)$, the corresponding asymptotic complexity of $\boldsymbol{\theta }_{q}$ is $O(polylog(n))$^23^, and this reduction in complexity significantly decreases the number of trainable parameters.

### Post-processing in QPG

Assuming we can accurately obtain the quantum state amplitudes or probabilities of a VQC, these values are restricted to a limited range due to the Born Rule^26^. Direct utilization of such constrained values would constrain the expressive power of the neural network. Consequently, an additional post-processing step is indispensable to map the VQC-derived quantum state amplitudes or probabilities to the value space of classical neural network weights in QPG. We compared three distinct strategies for this post-processing step in the relevant section, where these strategies specifically serve to mitigate the inherent domain mismatch between quantum outputs and the requirements of classical parameters.

The first approach is conceptually straightforward: it entails extracting the real part of a quantum state’s amplitude. This real part ranges over the interval $[-1, 1]$, thereby allowing for the representation of negative values. Accurately extracting this real part is easy in quantum simulators, yet it is challenging to implement on real quantum computers. It is due to the constraints imposed by the No-Cloning Theorem^30^. This approach is adopted in the first strategy, the details of which are provided in Eq. (3).

The second approach involves obtaining probabilities from measurements and transforming them into a broader value range. Probabilities are real numbers within $[0, 1]$ and must adhere to normalization conditions. However, as the quantum system scales, most elements’ values will approach zero, potentially leading to gradient vanishing in classical networks. To address this, the second strategy employs a linear projection using a classical Multilayer Perceptron (MLP) to map probabilities to values in $\mathbb {R}$, as described in Eq. (4).

The third strategy involves a shift operation and a scaling coefficient to transform probabilities into values within $[-\pi /4, \pi /4]$, as illustrated in Eq. (5).

Strategy 1: Real Component Extraction (RCE)

3 $$\begin{aligned} y_i = \textrm{Re}(\psi _i) \end{aligned}$$

The output $y_i$ is defined as the real component of the quantum amplitude $\psi _i$, where $\psi _i \in \mathbb {C}$ denotes the amplitude component of a normalized quantum state $|\psi \rangle$ in the orthonormal basis $\{|i\rangle \}$. Specifically, the quantum state $|\psi \rangle$ can be expanded as $|\psi \rangle = \sum _i \psi _i |i\rangle$, and it satisfies the normalization constraint $\sum _i |\psi _i|^2 = 1$ to ensure the conservation of total probability. It is worth noting that quantum states cannot be perfectly cloned due to the No-Cloning Theorem^30^, which makes the direct acquisition of quantum states challenging. However, quantum state tomography provides a viable approach to reconstruct high-fidelity approximate quantum states^31^, enabling effective extraction of amplitude information for practical applications.

Strategy 2: Linear Probability Projection (LPP)

4 $$\begin{aligned} y_i = \textbf{w}^\top \Big ( p_i \parallel \left( d_0, d_1, \ldots , d_k \right) \Big ) + b \end{aligned}$$

The output $y_i$ is generated by linearly projecting a concatenated vector composed of the quantum measurement probability $p_i \in [0,1]$ and the binary encoding of index $i$. Specifically, $[d_k \ldots d_0]_2 = i$ denotes the $(k+1)$-bit binary representation of $i$, with $d_0$ as the least significant bit. $\textbf{w} \in \mathbb {R}^n$ and $b \in \mathbb {R}$ are learnable parameters of a classical linear layer. The operator $\parallel$ signifies vector concatenation. This strategy combines probabilistic quantum information $p_i$ with discrete structural encoding of $i$, enhancing representational flexibility.

Strategy 3: Shift and Scale Transformation (SST)

5 $$\begin{aligned} y_i = \frac{\pi }{2} \left( p_i - 0.5 \right) \end{aligned}$$

The output $y_i$ maps the quantum measurement probability $p_i \in [0,1]$ to a number in $[-\pi /4,\pi /4]$. The transformation makes the negative weights possible.

All three post-processing methods have been experimentally validated, yet each presents distinct merits and drawbacks. Direct real-part extraction is conceptually straightforward and compatible with both positive and negative values; however, it is limited by a narrow expression range centered around zero and entails substantial challenges for physical hardware implementation. The LPP method features an extended value range and enables facile implementation on physical hardware, but it introduces a certain number of additional parameters. The SST method supports both positive and negative values without the introduction of extra parameters and allows for straightforward physical hardware deployment, though it similarly suffers from a narrow expression range concentrated near zero. In practical applications, method selection can be tailored to specific scenarios: LPP and SST are preferred for physical hardware implementations, SST is prioritized when precision is the key requirement, and LPP is chosen if minimizing the parameter count is the primary objective.

### Hybrid quantum classical training framework (HQC-TF)

**Fig. 2:**
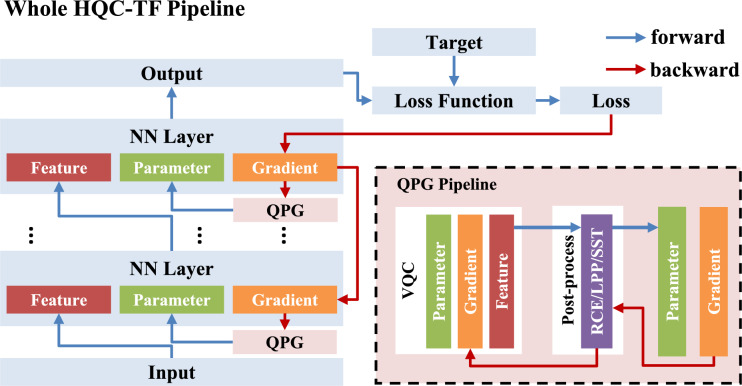
The Illustration of the HQC-TF Pipeline. The output of QPG modules serves as parameters for certain parts of the neural network, with the parameters of VQCs being trained instead of the parameters of those parts.

Previous Quantum-Train frameworks do not support the flexible selection of certain modules within a neural network for Quantum-Train while adopting classical training for other modules. To address this limitation, we propose the Hybrid Quantum-Classical Training Framework (HQC-TF). In HQC-TF, QPG can be flexibly applied in any Neural Network architecture by applying operation level integration, e.g., Conv2d, linear layer, or Multi-head attention. The complete set of parameters for the trained neural network consists of the QPG-generated parameters $\boldsymbol{\theta }_g$ and the classically learned parameters $\boldsymbol{\theta }_c$. Since $\boldsymbol{\theta }_c$ can be entirely generated by the VQC using its own parameters $\boldsymbol{\theta }_q$, it is optional to explicitly store $\boldsymbol{\theta }_c$. The process of HQC-TF is illustrated in Fig. 2. As illustrated in Fig. 2, HQC-TF operates through two core phases: in forward propagation, QPG first generates parameters for the integrated neural network layer, which are jointly computed with the layer’s input features to produce input features for the subsequent layer; in backward propagation, QPG’s parameters are adaptively updated based on backpropagated gradients.

**Algorithm 1:**
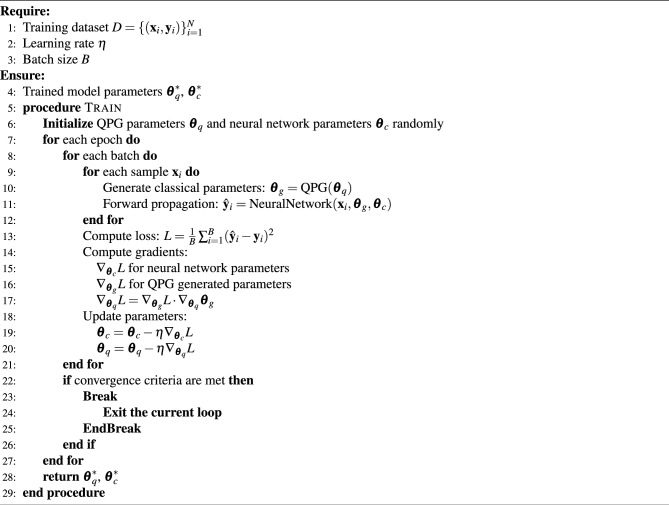
HQC-TF

In Algorithm 1, we elaborate on the computational process of the HQC-TF algorithm, in which the QPG is implemented via the quantum circuit illustrated in Fig. 1. Notably, the gradient computation for the quantum circuit within this algorithm is performed in a classical manner. By contrast, real quantum computers can compute gradients by leveraging the parameter shift rule^32^. This rule is a widely used method in quantum machine learning (QML) for calculating gradients of quantum circuit parameters: it works by shifting the values of these parameters and measuring the resulting differences in output. Such a mechanism makes it well-suited for implementation on real quantum computing hardware. Proper initialization is crucial to ensure effective training and convergence. Specifically, in this paper, the parameters of all our quantum circuits adopt random initialization with a uniform distribution over $$[0, 1)$$, while the parameters of the classical part use Kaiming initialization^33^. It is important to note that the quantum and classical parameters should not be initialized close to or equal to zero. Doing so may lead to gradient vanishing, a common issue in classical neural networks^33,34^.

### QPG trained convolution layers

**Fig. 3:**
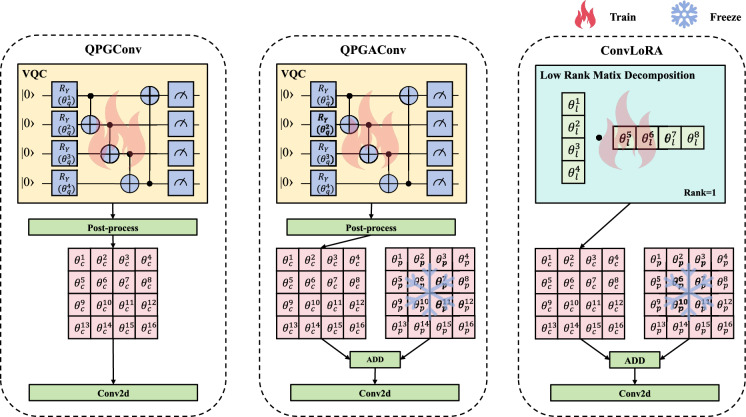
Illustration of the process of QPGConv, QPGAConv and ConvLoRA^21^. For 16 classic parameters, the minimum number of trainable parameters required for both QPGConv and QPGAConv is 4, while that for ConvLoRA^21^ is 8. $\theta _q$ = quantum parameter, $\theta _c$ = quantum generated parameter; $\theta _l$ = Low rank matrix decomposition parameter; $\theta _p$ = pre-trained parameter. The trainable parts and freeze parts are denoted with fire and snowflake icons respectively.

Since convolutions are widely used in OAR and tumor segmentation models, we proposed QPGConv and LoRA inspired QPGAConv for flexibility of integration, which are depicted in Fig. 3. The latter is an adapter version of the former. The mathematical descriptions of the original versions of QPGConv and QPGAConv are depicted in Eqs. (6) and (7), respectively.

6 $$\begin{aligned} W[c_{\text {in}}, c_{\text {out}}, k_h, k_w] = \text {VQC}_{c_{\text {out}}, k_h, k_w}(\phi _{c_{\text {out}}, k_h, k_w})[c_{\text {in}}] \end{aligned}$$

where *W* denotes the convolution kernel parameters; $c_{\text {in}} \in \{1, \dots , C_{\text {in}}\}$ and $c_{\text {out}} \in \{1, \dots , C_{\text {out}}\}$ are the indices of the input and output channels, respectively; $k_h \in \{1, \dots , K_h\}$ and $k_w \in \{1, \dots , K_w\}$ represent the height and width indices of the kernel; $\text {VQC}_{c_{\text {out}}, k_h, k_w}$ is the quantum neural network for generating parameters corresponding to output channel $c_{\text {out}}$ and kernel position $(k_h, k_w)$; and $\phi _{c_{\text {out}}, k_h, k_w}$ denotes its trainable parameters.

7 $$\begin{aligned} W'[c_{\text {in}}, c_{\text {out}}, k_h, k_w] = W[c_{\text {in}}, c_{\text {out}}, k_h, k_w] + \text {VQC}_{c_{\text {out}}, k_h, k_w}(\phi _{c_{\text {out}}, k_h, k_w})[c_{\text {in}}] \end{aligned}$$

where $W'$ represents the adapted convolution kernel parameters after incorporating quantum-generated adjustments; *W* is the base convolution kernel (e.g., pre-trained or initial kernel) consistent with Eq. (6); the notations $c_{\text {in}}, c_{\text {out}}, k_h, k_w$, $\text {VQC}_{c_{\text {out}}, k_h, k_w}$, and $\phi _{c_{\text {out}}, k_h, k_w}$ follow the same definitions as in Eq. (6); and the addition term denotes the quantum-generated adaptation increment for refining the base kernel *W*.

However, in real-world applications, QPG’s parameter efficiency can be significantly impaired when the input channel count is much smaller than the output channel count—for instance, in up-sampling scenarios. To address this limitation, we propose an optimized strategy tailored to enhance parameter utilization: specifically, we first compare the input and output channel counts, selecting the smaller value as the target channel number. For each target channel, a dedicated VQC is then utilized to generate the required parameters. Correspondingly, the mathematical formulations of QPGConv and QPGAConv are updated to Eqs. (8) and (9), respectively.

8 $$\begin{aligned} W[c_{\text {in}}, c_{\text {out}}, k_h, k_w] = {\left\{ \begin{array}{ll} \text {VQC}_{c_{\text {in}}, k_h, k_w}(\phi _{c_{\text {in}}, k_h, k_w})[c_{\text {out}}] & \text {if } C_{\text {in}} \le C_{\text {out}}, \\ \text {VQC}_{c_{\text {out}}, k_h, k_w}(\phi _{c_{\text {out}}, k_h, k_w})[c_{\text {in}}] & \text {if } C_{\text {in}} > C_{\text {out}}, \end{array}\right. } \end{aligned}$$

9 $$\begin{aligned} W'[c_{\text {in}}, c_{\text {out}}, k_h, k_w] = W[c_{\text {in}}, c_{\text {out}}, k_h, k_w] + {\left\{ \begin{array}{ll} \text {VQC}_{c_{\text {in}}, k_h, k_w}(\phi _{c_{\text {in}}, k_h, k_w})[c_{\text {out}}] & \text {if } C_{\text {in}} \le C_{\text {out}}, \\ \text {VQC}_{c_{\text {out}}, k_h, k_w}(\phi _{c_{\text {out}}, k_h, k_w})[c_{\text {in}}] & \text {if } C_{\text {in}} > C_{\text {out}}, \end{array}\right. } \end{aligned}$$

#### Illustrative example for channel indexing

To clarify the channel indexing logic in Eq. (8), we present two concrete scenarios using small, interpretable values below; the remaining formulas follow a similar logic, so we omit further elaboration.

Scenario 1: $C_{\text {in}} \ge C_{\text {out}}$ (Channel downsampling setting) Let $C_{\text {in}} = 512$, $C_{\text {out}} = 32$, $K_h = 2$, $K_w = 2$, correspond to a 2$\times$2 convolution kernel. Since $C_{\text {in}} \ge C_{\text {out}}$, the output channels have been selected as the target channels. One independent VQC is allocated for each target channel and kernel position, leading to a total of $32\times 2\times 2=128$ independent 9-qubit VQCs. For a specific kernel parameter *W*[4, 2, 1, 1] (input channel 4, output channel 2, kernel position $k_h=1, k_w=1$), the parameter is generated as:

$$W[4, 2, 1, 1] = \text {VQC}_{2, 1, 1}(\phi _{2, 1, 1})[4]$$.

Scenario 2: $C_{\text {in}} < C_{\text {out}}$ (Channel upsampling setting) Let $C_{\text {in}} = 64$, $C_{\text {out}} = 256$, $K_h = 3$, $K_w = 3$, correspond to a 3$\times$3 convolution kernel. Since $C_{\text {in}} < C_{\text {out}}$, the input channels have been selected as target channels. One independent VQC is allocated for each target channel and kernel position, resulting in a total of $64\times 3\times 3=576$ independent 8-qubit VQCs. For a specific kernel parameter *W*[1, 2, 1, 1] (input channel 1, output channel 2, kernel position $k_h=1, k_w=1$), the parameter is generated as:

$$W[1, 2, 1, 1] = \text {VQC}_{1, 1, 1}(\phi _{1, 1, 1})[2]$$.

## Experiment and result

The experiments were conducted on a single publicly available kidney dataset, which encompasses both organ segmentation and tumor segmentation tasks. These two tasks are distinct, as tumors are typically smaller and often exhibit irregular shapes compared to organs. Therefore, we evaluated the generalization capability of the proposed methods by conducting experiments on both tasks. All experiments were trained on the training set for 20 epochs, and the best results on the test set across these 20 epochs were used as the final results.

### Dataset

The dataset utilized in this study was sourced from the KiTS19 challenge^35^, a publicly available resource that comprises 300 de-identified clinical cases of patients who underwent partial or radical nephrectomy for kidney tumors between 2010 and 2018, of which 210 cases are publicly available. Urologic specialists conducted retrospective reviews of electronic medical records (EMR) and manually delineated renal capsules and tumor boundaries on multi-phase CT scans, following standardized protocols. Subsequent image processing involved Hounsfield Unit (HU) windowing (− 30 to 0) combined with adaptive noise reduction through the sequential application of $3 \times 3$ mean and $7 \times 7$ median filters. To ensure three-dimensional anatomical coherence, a hierarchical interpolation algorithm was systematically applied for axial slice-wise label propagation.Quality control was stringent, employing a dual-review framework with expert arbitration. Quantitative validation was conducted using a 30-case subset, which demonstrated exceptional inter-annotator agreement. This was evidenced by Dice Similarity Coefficients (DSC) of 0.983 for renal parenchyma and 0.923 for tumor regions. Among these 210 publicly available cases, the first 200 cases were selected for our study, while the remaining cases were excluded from our analysis. The KiTS19 challenge includes both organ and tumor segmentation labels, making it an ideal dataset for analyzing the effects of the QPG on both tasks.

#### Data of kidney segmentation

The cases were divided into training and test sets at a ratio of 9:1. Specifically, cases numbered 0 to 179 were assigned to the training set, while cases numbered 180 to 199 were allocated to the test set. The 3D images were processed into 2D slices, resulting in 14,871 2D images for the training set and 1,008 2D images for the test set. All images were resized to $256 \times 256$ pixels before being input into the models.

#### Data of kidney tumor segmentation

The data separation for kidney tumor segmentation followed the same procedure as for organ segmentation. The 3D images were also processed into 2D slices. For each image, we defined the Region of Interest (ROI) as the union of the kidney region and the tumor within it. We then cropped the tight bounding rectangular region of each ROI to create sub-images. This process yielded 4545 2D images for the training set and 384 2D images for the test set. Similar to the organ segmentation data, these images were resized to $256 \times 256$ pixels before being input into the models.

### Implementation details

The model training configuration of this study was built on a Python 3.9 environment, with PyTorch adopted as the core deep learning framework and TorchQuantum selected for the quantum simulation component. The experimental computing device was equipped with an Intel i9-9900K 3.60 GHz processor and a NVIDIA GeForce RTX 3090 graphics card with 24GB VRAM, accelerated by CUDA 12.4 and cuDNN 9.1 to enhance GPU computing performance. For data preprocessing, traditional libraries such as NumPy and OpenCV were utilized, while Scikit-learn and Matplotlib were leveraged for evaluation metric calculation and visualization. Regarding training parameters, the Adam optimizer was employed for optimization, configured with a learning rate of 0.001 and a weight decay of 5e−4, without a learning rate scheduler being utilized. The batch size was set to 8, and the number of training epochs was 20. In terms of backbone utilization strategies, the UNet, UNeXt, and TransUNet models did not adopt pre-trained backbones, whereas the UNetPP and MSNet models were trained based on pre-trained backbones.

### Evaluation metrics

The segmentation performance was evaluated using two widely adopted metrics: Intersection over Union (IoU) and DSC. For each test sample, we computed the sample-wise IoU and DSC, then the mean and standard deviation of these metrics across all test samples were calculated to comprehensively assess the model’s performance. The calculation of sample-wise IoU and Dice is shown in Eqs. (10) and (11).

10 $$\begin{aligned} \text {IoU}_i = \frac{|A_i \cap B_i|}{|A_i \cup B_i|}, \end{aligned}$$

11 $$\begin{aligned} \text {DSC}_i = \frac{2|A_i \cap B_i|}{|A_i| + |B_i|}. \end{aligned}$$

where $A_i$ and $B_i$ were the predicted and ground-truth segmentation masks for the $i$-th sample, then the mean and standard deviation were derived from these sample-wise metrics. In this paper, $\text {IoU}$ and $\text {DSC}$ refer to the means of the sample-specific $\text {IoU}_i$ and $\text {DSC}_i$, respectively. By calculating $\text {IoU}_i$ and $\text {DSC}_i$ for each sample, the contributions of samples with small target regions could be properly accounted for, as each sample had an equal weight in the final statistics. This approach avoided bias towards larger targets and ensured a robust assessment of segmentation consistency across all samples.

### Versatility experiment

In this study, we first conducted an experiment to evaluate the effects of classical training and the HQC-TF on five models for kidney and kidney tumor segmentation tasks. These models included UNet^36^, UNetPP^37^, UNeXt^38^, MSNet^39^, and TransUNet^9^. We used the IoU and DSC on the training set and test set as our comparison indicators. The detailed results are presented in Table 1, and the relevant calculation methods are described in the evaluation metrics section.

The training strategy was as follows: for all five models, the last convolution layer was trained using QPG, while the remaining parts were trained using classical methods. To distinguish the models trained with HQC-TF, we added the prefix ‘Q’ to their original names (e.g., QUNetPP). For each model trained with HQC-TF, we compared three post-processing methods–RCE, LPP, and SST.

**Fig. 4:**
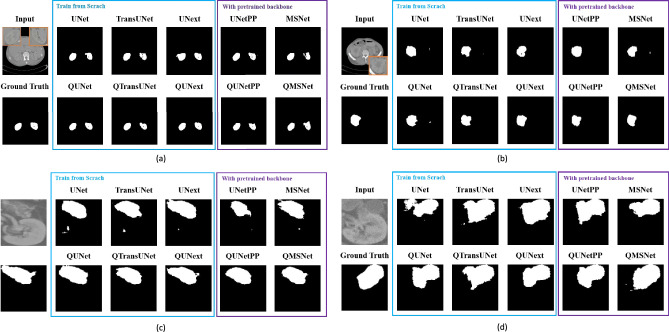
Illustration of effects of HQC-TF on different models. It can be found that the models trained with HQC-TF exhibited a significant improvement in the segmentation performance of kidneys and kidney tumors compared to classically trained models, particularly for kidney tumor segmentation.

To intuitively demonstrate the impact of HQC-TF, we performed a visualization analysis. We selected two samples from the kidney and kidney tumor datasets, respectively, to visually compare the segmentation results of the five models under both pure classical training and HQC-TF. The visualization results are shown in Fig. 4. Specifically, the areas within the yellow boxes in the input images of Figs. 4a and 4b are enlarged views of the tumor regions.

#### Quantitative results

**Table 1:** Performance comparison of full parameters training with different configurations. PPS, Post-processing strategy; RCE, Real component extraction; LPP, Linear probability projection; SST, Shift and scale transformation. Bold indicates the best result in the group; Bold + underline indicates the overall best result.

Model	PPS	Kidney				Kidney tumor			
		Train		Test		Train		Test	
		IoU	DSC	IoU	DSC	IoU	DSC	IoU	DSC
Trained from scratch									
UNet	–	0.8539	0.9196	0.8004	0.8623	0.8218	0.8791	0.6031	0.6959
QUNet	RCE	0.8550	0.9233	0.8012	0.8652	0.8222	0.8805	0.6014	0.6966
	LPP	**0.8617**	**0.9295**	**0.8154**	**0.8701**	**0.8393**	**0.9008**	**0.6271**	**0.7249**
	SST	0.8602	0.9268	0.8060	0.8687	0.8325	0.8955	0.6138	0.7134
UNeXt	–	0.8769	0.9336	0.8151	0.8807	0.8514	0.9023	0.6149	0.7061
QUNeXt	RCE	**0.8850**	**0.9412**	**0.8225**	**0.8863**	0.8546	0.9057	0.6274	0.7097
	LPP	0.8813	0.9395	0.8212	0.8856	**0.8574**	**0.9069**	0.6184	**0.7128**
	SST	0.8802	0.9338	0.8213	0.8851	0.8530	0.9049	**0.6299**	0.7123
TransUNet	–	0.9460	0.9689	0.8761	0.9028	0.7482	0.8531	0.5764	0.6700
QTransUNet	RCE	0.9457	0.9685	0.8760	0.9028	0.7546	0.8551	0.5903	0.6787
	LPP	**0.9503**	**0.9735**	**0.8825**	**0.9063**	**0.7699**	**0.8636**	**0.5983**	**0.6859**
	SST	0.9482	0.9714	0.8774	0.9052	0.7495	0.8545	0.5865	0.6738
With pre-trained backbone									
UNetPP	–	0.9479	0.9721	0.8776	0.9067	0.8261	0.8816	0.6274	0.7244
QUNetPP	RCE	0.9268	0.9564	0.8653	0.9015	0.8317	0.9010	0.6569	0.7341
	LPP	0.9485	0.9745	0.8791	0.9075	**0.8444**	**0.9183**	**0.6699**	**0.7468**
	SST	**0.9514**	**0.9787**	**0.8814**	**0.9099**	0.8309	0.8914	0.6505	0.7305
MSNet	–	0.8622	0.9409	0.8108	0.8817	0.6726	0.7516	0.6700	0.7472
QMSNet	RCE	0.8615	0.9381	0.8133	0.8825	0.6750	0.7523	0.6773	0.7500
	LPP	0.8612	**0.9443**	**0.8192**	**0.8897**	**0.6799**	**0.7591**	**0.6827**	**0.7551**
	SST	**0.8694**	0.9426	0.8112	0.8834	0.6773	0.7526	0.6714	0.7505

The results of the test set clearly demonstrate that HQC-TF enhances the performance of both kidney and kidney tumor segmentation, with more notable improvements in kidney tumor segmentation.

Kidney segmentation HQC-TF yields consistent gains on the test set of kidney segmentation across all models. Among scratch-trained models, QUNet (with LPP) outperformed UNet by 1.87% in IoU (0.8154 vs. 0.8004) and 0.90% in DSC (0.8701 vs. 0.8623). For pre-trained models, QMSNet (with SST) showed a 1.03% IoU improvement (0.8192 vs. 0.8108) and a 0.90% DSC improvement (0.8897 vs. 0.8817) over MSNet.

Kidney tumor segmentation The benefits of HQC-TF were more pronounced on the kidney tumor segmentation test set. For scratch-trained models, QUNeXt (with SST) outperformed UNeXt by 2.44% in IoU (0.6299 vs. 0.6149), while QTransUNet (with LPP) improved TransUNet’s IoU by 3.79% (0.5983 vs. 0.5764). For pre-trained models, QMSNet (with LPP) achieved the highest test IoU of 0.6827 among all models, 1.89% higher than MSNet (0.6700), and a DSC of 0.7551, 1.06% higher than MSNet (0.7472). Notably, QUNetPP (with LPP) reached an IoU of 0.6699, 6.77% higher than UNetPP (0.6274), and a DSC of 0.7468, 3.09% higher than UNetPP (0.7244).

Comparison of training and test results reveals that HQC-TF enhanced both the model’s fitting capability (on the training set) and generalization performance (on the test set). Specifically, in terms of performance on the training set of kidney segmentation: QTransUNet (with LPP) increased the IoU of TransUNet by 0.45% (0.9503 vs. 0.9460); meanwhile, QUNetPP (with SST) achieved the highest training IoU (0.9514) among pre-trained models, which was 0.37% higher than that of UNetPP. In terms of performance on the training set of kidney tumor segmentation: QUNet (with LPP) raised the IoU of UNet by 2.13% (0.8393 vs. 0.8218); additionally, QUNetPP (with LPP) outperformed UNetPP by 2.21% in IoU (0.8444 vs. 0.8261). The results in the training set and test set collectively confirmed the positive effect of HQC-TF on mitigating model overfitting.

Regarding post-processing strategies, LPP consistently delivered excellent performance across most models and tasks. On the test set for kidney segmentation, LPP performs the best among 3 out of the 5 models; on the test set for kidney tumor segmentation, LPP performs the best across all models.

#### Qualitative results

Kidney segmentation Figure 4a,b show two kidney segmentation samples. It was observed that the masks predicted by purely classical-trained models exhibited inconsistent shapes compared to the annotations and generated some noise. In contrast, the masks predicted by HQC-TF trained models were more consistent with the true annotations in shape, and noise generation was alleviated. Specifically, in Fig. 4a, both UNetPP and MSNet produced obvious noise separated from the tumor region, while QUNetPP and QMSNet did not generate such noise. In Fig. 4b, MSNet and TransUNet both produced noise separated from the tumor region, while QMSNet and QTransUNet did not. UNext generated hollow noise inside the tumor, but QUNext did not. Although both QUNet and UNet produced noise separated from the tumor region, the overall segmentation effect of QUNet was still better than that of UNet.

Kidney tumor segmentation Figure 4c,d show two kidney tumor segmentation samples. It could be observed that the masks predicted by classical-trained models have larger deviations from the annotations and significantly more noise. In contrast, the masks obtained by HQC-TF were more consistent with the true annotations in shape, and the noise was significantly reduced. Specifically, in Fig. 4c, all purely classical-trained models generated noise that was separated from the kidney region, while HQC-TF trained models did not produce such errors. The mask shape predicted by UNetPP had a large deviation from the true annotation, while the mask shape predicted by QUNetPP was more consistent with the true annotation. In Fig. 4d, both UNet and TransUNet generated noise that was separated from the kidney region, while QUNet and QTransUNet did not. The difference in mask shape between all classical-trained models and the true annotations was larger than that of HQC-TF trained models, especially for UNet, which showed obvious errors. Although QMSNet generates noise that was separated from the kidney region while MSNet did not, the overall segmentation effect of QMSNet was still better than that of MSNet.

### Scalability experiment

**Fig. 5:**
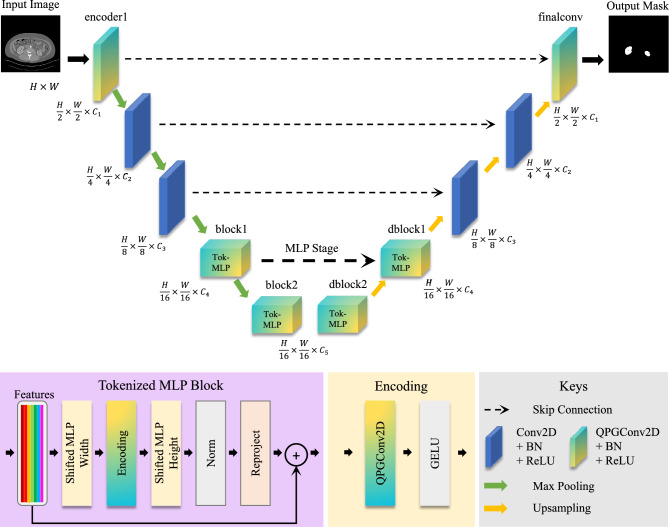
The Illustration of UNext Trained with HQC-TF. QPGConv2D is 2D QPGConv. The structure of UNext was maintained and the blocks with blue and yellow gradient colored denoted the parameter of the blocks were generated through QPG.

To validate the efficacy of the HQC-TF method across all architectural components of a model and to explore how incrementally increasing the number of QPG modules impacts model training, we gradually expanded the QPG module count from 2 to 5 and integrated these QPG modules into distinct architectural components of the classical UNext network. We adopted the UNext model as the baseline architecture; its structure is illustrated in Fig. 5, where yellow-green gradient coloring marks the regions where QPG modules are deployed to train the corresponding classical network components. Detailed descriptions of these integration schemes are provided in Table 2 and Fig. 5. It can be observed that the deployed QPG modules span the front, middle, and rear segments of the model and encompass both downsampling and upsampling stages, thereby enabling a comprehensive assessment of HQC-TF’s applicability across distinct spatial positions in the model architecture, as well as the impact of an increasing number of implemented QPG modules on the training of classical neural network components.

**Table 2:** Scheme description.

Scheme	Components
1	Encoder1, finalconv
2	Encoder1, block2, finalconv
3	Encoder1, block2, dblock2
4	Block1, block2
5	Block1, block2, dblock1
6	Block1, block2, dblock2
7	Block1, block2, dblock1, dblock2
8	Block1, block2, dblock1, dblock2, finalconv
9	Block2, finalconv
10	Block2, dblock2, finalconv
11	Block2, dblock1, dblock2, finalconv

**Fig. 6:**
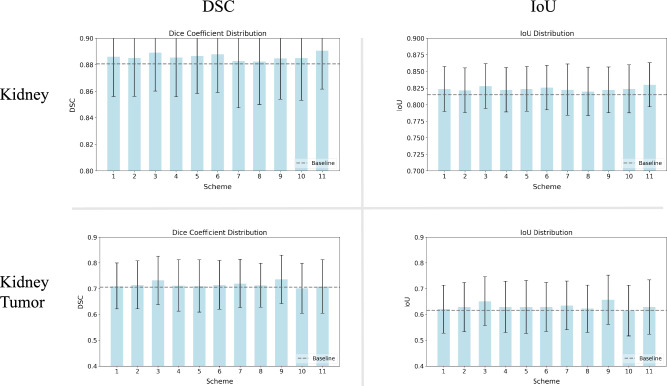
The scalability of HQC-TF. It could be found that applying 2 to 5 QPGConv modules to different regions of the model almost universally improved the model performance.

As shown in Fig. 6, an analysis of the Dice Similarity Coefficient and Intersection over Union metrics across eleven organ segmentation schemes confirms that HQC-TF outperforms classical training in most scenarios. For kidney segmentation, Schemes 3, 6, and 11 achieve optimal performance, while all eleven schemes surpass pure classical training. The performance gains yielded by HQC-TF in kidney segmentation are modest yet consistently reliable. In contrast, kidney tumor segmentation performance varies across schemes. Schemes 3, 7, and 9 lead in performance, with Schemes 3 and 9 demonstrating statistically significant superiority over pure classical training. Scheme 10 stands as the sole case where quantum-classical training marginally underperforms classical methods.

Notably, Schemes 3, 6, 7, 9, and 11 incorporate 2 to 4 QPG modules and span the early, middle, and late stages of the model. This reveals that increasing the number of QPG modules does not exert a significant impact on performance while validating HQC-TF’s effectiveness across all segments of the model. Collectively, HQC-TF delivers more pronounced improvements in kidney tumor segmentation, albeit with a minor risk of performance degradation in specific schemes, such as Scheme 10.

### Hyperparameter experiment

**Fig. 7:**
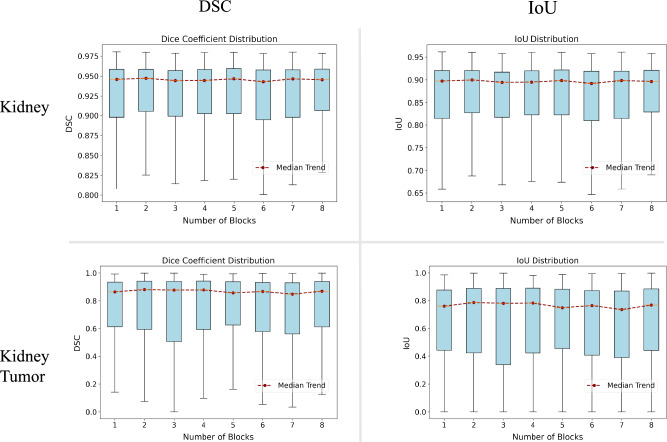
The impact of quantum parameter quantity on the performance of HQC-TF. It could be found that the number of variational layers had little impact on the improvement of model performance, which indicated that shallow circuits are sufficient.

While studies have demonstrated that over-parameterization aids VQCs in finding global optima^40^, there is currently no evidence to support its benefits for optimizing hybrid quantum-classical networks. The determination of the optimal number of parameters in such hybrid networks largely depends on empirical findings. To explore the impact of quantum parameter size on the overall performance of the HQC-TF, we designed a set of scaling experiments that varied the number of blocks. The QUNext model was chosen as the experimental model. The results of these experiments are presented in Fig. 7.

In organ segmentation tasks, as the number of repeated blocks increased, the median trend line of the DSC exhibited minor fluctuations but generally stabilized and approached 0.95. This indicates that the model maintains consistent performance in organ segmentation. Similarly, the median trend line of the IoU remains highly stable, hovering around 0.9.

In kidney tumor segmentation tasks, similar trends were observed. The DSC for tumors also showed remarkable stability, with its median trend line consistently approaching 0.9. Meanwhile, the IoU distribution for tumors maintained comparable stability, with its median trend line remaining at approximately 0.8.

These results demonstrated that the performance of HQC-TF was not significantly affected by variations in the number of QPG parameters. Notably, even at lower parameter levels, such as with just one block, the QPG remained sufficiently effective.

### Comparative experiments

To compare the performance differences between HQC-TF and current mainstream schemes during the training process of kidney segmentation models, we designed comparative experiments under three distinct training scenarios, where the VGG backbone network, pre-trained on the ImageNet dataset, was adopted consistently across all scenarios. For the full-parameter training scenario, the entire network was subjected to training, with all parameters updated throughout the training process. In the partial-parameter training scenario, the parameters of the backbone network were frozen, and only the parameters of the non-backbone network were trained. The third scenario focused on parameter-efficient fine-tuning: parameter-efficient fine-tuning was applied to the backbone network, while the non-backbone network was trained using conventional methods.

In line with the three training scenarios outlined above, three comparative experiments were developed for the UNetPP model to assess performance variations. In the first comparative experiment, the final layer of UNetPP was trained using classical training and quantum training, respectively, while all other components of the network were trained through classical training. For the second comparative experiment, the parameters of the backbone network of UNetPP were frozen, and all other settings remained consistent with those of the first experiment. As for the third comparative experiment, it involved a performance comparison between the mainstream LoRA based method ConvLoRA^21^ and our proposed LoRA inspired QPGAConv; specifically, the second layer of the backbone network was trained using classical training and quantum training, respectively, while the remaining layers of the backbone network and all non-backbone network layers were trained through classical training.

**Table 3:** Comparison of performance UNetPP under different training It could be found that HQC-TF was generally effective under three training settings. When compared with ConvLoRA, although ConvLoRA achieved better performance at rank=16, HQC-TF not only outperformed ConvLoRA at rank=8 but also had a smaller number of parameters.

Architecture	Setting	Framework	Train IOU	Train DSC	Test IOU	Test DSC	Params
UNetPP	Train all parameters	Classic	0.9479	0.9721	0.8776	0.9067	576
		HQC-TF	**0.9485**	**0.9745**	**0.8791**	**0.9075**	**108**
	Freeze backbone	Classic	0.9106	0.9514	0.8522	0.9025	576
		HQC-TF	**0.9181**	**0.9526**	**0.8534**	**0.9049**	**108**
	PEFT	Classic	0.9106	0.9514	0.8522	0.9025	36864
		ConvLoRA(r=8)	0.9257	0.9585	0.8526	0.9035	9216
		ConvLoRA(r=16)	**0.9307**	**0.9621**	**0.8652**	**0.9148**	18432
		HQC-TF	0.9275	0.9596	0.8549	0.9058	**6912**

As shown in Table 3, in the scenarios of full-parameter training and backbone freezing, HQC-TF outperforms the classic framework in both the training metrics Train IOU and Train DSC, as well as the test metrics Test IOU and Test DSC. Meanwhile, it significantly reduces the number of parameters. Specifically, in full-parameter training, HQC-TF requires only 108 parameters, which is far fewer than the 576 parameters needed by the Classic framework, fully demonstrating superior performance and parameter efficiency.

In the PEFT scenario, the performance of ConvLoRA gradually improves as its rank increases from 8 to 16; yet, this improvement comes at the cost of increased parameters. In contrast, HQC-TF achieves performance that falls between ConvLoRA with a rank of 8 and ConvLoRA with a rank of 16, while using only 6912 parameters—much fewer than the 18432 parameters required by ConvLoRA with a rank of 16–thus achieving a better balance between performance and parameter overhead. Overall, HQC-TF exhibits the advantage of ”enhancing performance while significantly reducing parameter scale” across different training scenarios, and its ability to balance performance and parameter overhead is particularly prominent in the PEFT scenario.

## Discussion

The results from both the quantitative and qualitative analyzes sections clearly demonstrated that the HQC-TF significantly enhanced the segmentation performance for both kidney and kidney tumor, with particularly notable improvements in the more challenging task of kidney tumor segmentation. This improvement holds special clinical significance, as tumor segmentation is more challenging than OAR segmentation, and current deep learning models still fail to perform well in this task. The findings suggested that HQC-TF can effectively address this critical gap.

The scalability experiments further revealed that QPG is not only effective in specific areas but also across the entire segmentation network. This capability boosts confidence for future large-scale experiments and underscores the potential for broader application. In terms of quantum parameter count, the results indicated that even a shallow quantum circuit can achieve excellent performance, yet no significant improvement was observed with an increase in the number of layers. On the positive side, this is promising for implementation on resource-constrained quantum computers in the NISQ era; on the negative side, it may lead to limited performance gains. The performance improvement achieved by shallow circuits may stem from the adequate expressive power of shallow circuits for the present scenario, or from the synergistic enhancement between classical training and quantum training within the HQC-TF training framework. In contrast, deeper variational quantum circuits, or VQCs, fail to yield further performance improvements, likely due to the training challenges they encounter in quantum computing implementations, such as barren plateaus^41^. Comparative experiments demonstrated the effectiveness of HQC-TF under different training configurations. Experiments using QPGAConv showed that HQC-TF can deliver effective performance with fewer parameters compared with ConvLoRA.

In neural networks, channel independence is of great significance, as exemplified by the necessity of head independence in multi-head attention^24^ and kernel independence in convolution layers^25^ for maintaining the independence of different feature channels. For the HQC-TF framework, it adopts independent VQCs to generate parameters for different channels. This design not only effectively achieves channel independence but also concomitantly realizes weight normalization^42^. Importantly, this inherent weight normalization eliminates the need for explicit weight normalization computations, thereby reducing redundant calculation overhead. Although the post-processing of QPG might disrupt the weight normalization, the framework still accomplishes this normalization computation in the intermediate process and this intermediate-stage realization is presumably meaningful in practical scenarios.

Many dense encoding methods require decomposition into very long circuits with numerous control gates, and they generally exhibit superlinear scaling with the amount of data. This makes it nearly impossible to scale up dense encoding in the NISQ era on real quantum machines. However, QPG does not require encoding–It also does not impose a clear lower bound on circuit depth. The depth of QPG circuits can be flexibly selected according to specific problems, thus offering new perspectives for solving large-scale real-world problems in the NISQ era.

### The limitations and future outlook

Despite demonstrating the effectiveness of HQC-TF in multiple kidney and kidney tumor segmentation models, our work still has several limitations. First, the requirement for substantial classical computational resources to perform quantum simulations limits the scale of our experiments. Future research can explore methods to reduce these classical computational demands, thereby enabling the validation of HQC-TF at larger scales. Second, the application scope of HQC-TF in this study is restricted to kidney and kidney tumor segmentation models; it has not been extended to segmentation tasks for other OARs or tumor types. In fact, the design logic of HQC-TF is not limited to the aforementioned scenarios. It can be fully generalized to segmentation models for other OARs and tumors, and further possesses application potential in any deep neural network that incorporates convolutional layers. Third, this study did not conduct multi-center or cross-modal experiments. Subsequent research can further investigate the generalization performance of HQC-TF across different datasets and modalities. Finally, this paper primarily focuses on experimental research. Future studies could delve into theoretical aspects, such as analyzing HQC-TF from the perspective of learning theory.

## Conclusion

In conclusion, the proposed HQC-TF achieves significantly better performance than classical training methods in both OAR segmentation and tumor segmentation. Its channel-independent design not only ensures inter-channel independence but also enables weight regularization. Experimental results from training on five mainstream models demonstrate that HQC-TF possesses excellent versatility. Furthermore, results from applying QPG to different modules of the UNext model demonstrate that HQC-TF has good scalability. In addition, experimental results for QPGConv and QPGAConv show that HQC-TF has outstanding flexibility and exhibits advanced performance in various training scenarios. The effectiveness of shallow circuits positions HQC-TF as a promising quantum algorithm in the NISQ era.

## Data Availability

The dataset used in this study is publicly available from the 2019 KiTS Challenge repository at https://github.com/neheller/kits19.

## References

[1] Liu, X. et al. Towards more precise automatic analysis: A systematic review of deep learning-based multi-organ segmentation. *Biomed. Eng. Online* **23**, 52. 10.1186/s12938-024-01238-8 (2024). 38851691 10.1186/s12938-024-01238-8 PMC11162022

[2] Wang, X. et al. A pathology foundation model for cancer diagnosis and prognosis prediction. *Nature* **634**, 970–978. 10.1038/s41586-024-07894-z (2024). 39232164 10.1038/s41586-024-07894-z PMC12186853

[3] Teo, Z. L. et al. Generative artificial intelligence in medicine. *Nat. Med.* **31**, 3270–3282. 10.1038/s41591-025-03983-2 (2025). 41053447 10.1038/s41591-025-03983-2

[4] Muksimova, S., Umirzakova, S., Baltayev, J. & Cho, Y.-I. Rl-cervix. net: a hybrid lightweight model integrating reinforcement learning for cervical cell classification. *Diagnostics* **15**, 364. 10.3390/diagnostics15030364 (2025). 39941293 10.3390/diagnostics15030364 PMC11816595

[5] Ye, X. et al. Comprehensive and clinically accurate head and neck cancer organs-at-risk delineation on a multi-institutional study. *Nat. Commun.* **13**, 6137. 10.1038/s41467-022-33178-z (2022). 36253346 10.1038/s41467-022-33178-z PMC9576793

[6] Jin, S. et al. Gswinclnet: a robust framework for brain tumor segmentation via shifted window attention and cross-scale fusion. *Sci. Rep.*10.1038/s41598-025-31937-8 (2025). 41388080 10.1038/s41598-025-31937-8 PMC12808724

[7] Zhu, J., Hamdi, A., Qi, Y., Jin, Y. & Wu, J. Medical sam 2: Segment medical images as video via segment anything model 2. https://arxiv.org/abs/2408.00874] (2024).

[8] Wu, J. et al. Medsegdiff: Medical image segmentation with diffusion probabilistic model. *In MIDL* **227**, 1623–1639 (2023).

[9] Chen, J. et al. Transunet: Rethinking the u-net architecture design for medical image segmentation through the lens of transformers. *MIA* **97**, 103280. 10.1016/j.media.2024.103280 (2024). 10.1016/j.media.2024.103280 39096845

[10] Ma, J. et al. Segment anything in medical images. *Nat. Commun.* **15**, 654. 10.1038/s41467-024-44824-z (2024). 38253604 10.1038/s41467-024-44824-z PMC10803759

[11] Chen, T., Liang, L., Ding, T., Zhu, Z. & Zharkov, I. Otov2: Automatic, generic, user-friendly. https://arxiv.org/abs/2303.06862 (2023).

[12] Gou, J., Yu, B., Maybank, S. J. & Tao, D. Knowledge distillation: A survey. *IJCV* **129**, 1789–1819. 10.1007/s11263-021-01453-z (2021).

[13] Krishnamoorthi, R. Quantizing deep convolutional networks for efficient inference: A whitepaper. https://arxiv.org/abs/1806.08342 (2018).

[14] Houlsby, N. et al. Parameter-efficient transfer learning for nlp. *ICML*. 2790–2799 (2019).

[15] Xin, Y. et al. Parameter-efficient fine-tuning for pre-trained vision models: A survey and benchmark. https://arxiv.org/abs/2402.02242 (2025).

[16] Liu, W., Shen, X., Pun, C.-M. & Cun, X. Explicit visual prompting for low-level structure segmentations. *CVPR*. 19434–19445. 10.1109/cvpr52729.2023.01862 (2023).

[17] Li, X. L. & Liang, P. Prefix-tuning: Optimizing continuous prompts for generation. *ACL*. 4582–4597. 10.18653/v1/2021.acl-long.353 (2021).

[18] Zhang, J. O., Sax, A., Zamir, A., Guibas, L. & Malik, J. Side-tuning: a baseline for network adaptation via additive side networks. *ECCV*. 698–714. 10.1007/978-3-030-58580-8_41 (2020).

[19] Hu, E. J. et al. Lora: Low-rank adaptation of large language models. *ICLR* **1**, 3 (2022).

[20] Zhong, Z., Tang, Z., He, T., Fang, H. & Yuan, C. Convolution meets lora: Parameter efficient finetuning for segment anything model. https://arxiv.org/abs/2401.17868 (2024).

[21] Aleem, S., Dietlmeier, J., Arazo, E. & Little, S. Convlora and adabn based domain adaptation via self-training. *ISBI*. 1–5. 10.1109/isbi56570.2024.10635661 (2024).

[22] Liu, C.-Y. et al. Training classical neural networks by quantum machine learning. *QCE* **2**, 34–38 (2024).

[23] Liu, C.-Y. et al. Quantum-train: Rethinking hybrid quantum-classical machine learning in the model compression perspective. *Quantum Mach. Intell.* **7**. 10.1007/s42484-025-00305-0 (2025).

[24] Vaswani, A. et al. Attention is all you need. NeurIPS 30. https://dl.acm.org/doi/10.5555/3295222.3295349 (2017).

[25] Zhao, X. et al. A review of convolutional neural networks in computer vision. *Artif. Intell. Rev.* **57**, 99. 10.1007/s10462-024-10721-6 (2024).

[26] Neumaier, A. The born rule—100 years ago and today. *Entropy* **27**10.3390/e27040415 (2025). 10.3390/e27040415 PMC12026146 40282650

[27] Chen, S., Cotler, J., Huang, H.-Y. & Li, J. The complexity of nisq. *Nat. Commun.* **14**, 6001. 10.1038/s41467-023-41217-6 (2023). 37752125 10.1038/s41467-023-41217-6 PMC10522708

[28] AbuGhanem, M. Ibm quantum computers: Evolution, performance, and future directions. *J. Supercomput.* **687**, 10.1007/s11227-025-07047-7 (2024).

[29] AI, G. Q. Suppressing quantum errors by scaling a surface code logical qubit. *Nature* **614**, 676–681. 10.1038/s41586-022-05434-1 (2023). 10.1038/s41586-022-05434-1 PMC9946823 36813892

[30] Wootters, W. K. & Zurek, W. H. A single quantum cannot be cloned. *Nature* **299**, 802–803. 10.1038/299802a0 (1982).

[31] Hu, C.-K. et al. Experimental sample-efficient quantum state tomography via parallel measurements. *PRL* **133**, 160801. 10.1103/PhysRevLett.133.160801 (2024). 10.1103/PhysRevLett.133.160801 39485955

[32] Wierichs, D., Izaac, J., Wang, C. & Lin, C. Y.-Y. General parameter-shift rules for quantum gradients. *Quantum* **6**, 677. 10.22331/q-2022-03-30-677 (2022).

[33] He, K., Zhang, X., Ren, S. & Sun, J. Delving deep into rectifiers: Surpassing human-level performance on imagenet classification. *ICCV*. 1026–1034. 10.1109/ICCV.2015.123 (2015).

[34] Glorot, X., Bordes, A. & Bengio, Y. Deep sparse rectifier neural networks. *AISTATS*. 315–323 (2011).

[35] Heller, N. et al. The kits19 challenge data: 300 kidney tumor cases with clinical context, ct semantic segmentations, and surgical outcomes. https://arxiv.org/abs/1904.00445 (2020).

[36] Ronneberger, O., Fischer, P. & Brox, T. U-net: Convolutional networks for biomedical image segmentation. *MICCAI*. 234–241. 10.1007/978-3-319-24574-4_28 (2015).

[37] Zhou, Z., Rahman Siddiquee, M. M., Tajbakhsh, N. & Liang, J. Unet++: A nested u-net architecture for medical image segmentation. *DLMIA ML-CDS*. 3–11. 10.1007/978-3-030-00889-5_1 (2018). 10.1007/978-3-030-00889-5_1 PMC7329239 32613207

[38] Valanarasu, J. M. J. & Patel, V. M. Unext: Mlp-based rapid medical image segmentation network. *MICCAI*. 23–33. 10.1007/978-3-031-16443-9_3 (2022).

[39] Zhao, X., Zhang, L. & Lu, H. Automatic polyp segmentation via multi-scale subtraction network. *MICCAI*. 120–130. 10.1007/978-3-030-87193-2_12 (2021).

[40] Abbas, A. et al. The power of quantum neural networks. *Nat. Comput. Sci.* **1**, 403–409. 10.1038/s43588-021-00084-1 (2021). 38217237 10.1038/s43588-021-00084-1

[41] McClean, J. R., Boixo, S., Smelyanskiy, V. N., Babbush, R. & Neven, H. Barren plateaus in quantum neural network training landscapes. *Nat. Commun.* **9**, 4812. 10.1038/s41467-018-07090-4 (2018). 30446662 10.1038/s41467-018-07090-4 PMC6240101

[42] Salimans, T. & Kingma, D. P. Weight normalization: A simple reparameterization to accelerate training of deep neural networks. *NeurIPS* **29**, 901–909 (2016).

